# Optimal Intracorporeal Anastomosis for Colectomy: A Comparative Experimental Evaluation Using 3D Anastomosis Models

**DOI:** 10.1111/ases.70048

**Published:** 2025-03-17

**Authors:** Yoshiaki Fujii, Seiya Yamamoto, Sho Kimura, Shogo Suzuki, Hirotaka Miyai, Hiroki Takahashi, Yoichi Matsuo, Kenji Kobayashi, Shuji Takiguchi

**Affiliations:** ^1^ Department of Gastroenterological Surgery Nagoya City University Graduate School of Medical Sciences Nagoya Japan; ^2^ Department of Surgery Kariya Toyota General Hospital Kariya Aichi Japan

**Keywords:** anastomosis, imaging, patency, pathologic, surgical, three‐dimensional

## Abstract

**Introduction:**

Application of intracorporeal anastomosis is gradually becoming widespread; however, there are no detailed reports on its configuration. We aimed to create three‐dimensional intracorporeal anastomosis models and compare their configurations in detail.

**Methods:**

Three types of intracorporeal anastomosis models were used: overlap anastomosis, delta‐shaped anastomosis, and functional end‐to‐end anastomosis. In experiment 1, three‐dimensional images of each anastomosis model were created. Additionally, the length of each staple line comprising the anastomotic site was measured. In experiment 2, the lengths of intestinal mobilization required for different anastomoses were compared.

**Results:**

The circumference of the anastomosis in overlap anastomosis (141.5 ± 3.3 mm) was significantly greater than that in delta‐shaped anastomosis (87.9 ± 0.9 mm; *p* < 0.001) and functional end‐to‐end anastomosis (89.6 ± 10 mm; *p* < 0.0001). The length of the intestinal tract after anastomosis in delta‐shaped anastomosis (33 ± 6.9 mm) was significantly shorter than that in functional end‐to‐end anastomosis (76 ± 2 mm; *p* < 0.0001) and overlap anastomosis (60 ± 5 mm; *p* < 0.002).

**Conclusions:**

We successfully constructed three‐dimensional images of intracorporeal anastomosis models. These results suggest that overlap anastomosis led to the formation of the largest anastomotic site, while minimal bowel mobilization was required in the delta‐shaped anastomosis.

## 1 Introduction

The optimal type of intracorporeal anastomosis (IA) remains unclear. This is due to the inability to objectively compare the characteristics of different types of anastomoses, including their advantages and disadvantages. As robotic surgery becomes more prevalent, the frequency of IA in minimally invasive colectomies has increased [1]. Meta‐analyses and prospective clinical trials comparing the short‐term outcomes of IA and extracorporeal anastomosis (EA) have suggested that the former offers significant benefits owing to its minimally invasive nature. Notably, these advantages include reduced postoperative pain, decreased wound infections, earlier recovery of gastrointestinal function, and lower incidence of incisional hernias associated with the Pfannenstiel incision [1, 2, 3, 4, 5, 6, 7, 8]. Additionally, the mid‐to‐long‐term outcomes of IA are comparable with those of EA [9, 10, 11]. Generally, IA in colectomy is performed via side‐to‐side anastomosis (overlap anastomosis [OLA] and functional end‐to‐end anastomosis [FEEA]) or end‐to‐end anastomosis (delta‐shaped anastomosis [DSA]) using linear stapler [12, 13, 14, 15]. Further innovations in these techniques have been introduced to maximize the effective use of linear staplers and minimize hand‐sewing [12]. Thus, IA in colectomies is gradually becoming more common; however, there is currently no evidence‐based research to guide the selection of the optimal anastomosis technique. The objective of this study was to create three‐dimensional models of anastomoses based on three representative totally stapled IA techniques, compare and analyze their configurations in detail using objective data, and evaluate the characteristics of each technique. This study could potentially assist in selecting the most appropriate IA procedure based on the intraoperative conditions encountered during colectomy.

## 2 Materials and Methods

### 2.1 Materials

Fresh pig colons weighing 100–120 kg were used in this study. Specimens were obtained from animals that were killed for use in approved non‐gastrointestinal research studies. The specimens were used within 48 h of death. Each segment of the colonic tract was approximately 20 cm long and 3.5–4 cm wide. This study was approved by the Ethics Committee of the Kariya Toyota General Hospital (approval number: 658) and conducted in compliance with the ARRIVE guidelines. Additionally, all methods were performed in accordance with relevant guidelines and regulations.

### 2.2 Experimental Procedures

We created three specimens for each of the three different types of intracorporeal anastomotic models, including two types of side‐to‐side anastomosis (OLA and FEEA) and one type of end‐to‐end anastomosis (DSA). All procedures for each anastomosis were performed by the same surgeon using forceps (rather than hands) and following the intracorporeal anastomotic process. The stapling device used was the ECHELON FLEX GST System 60 GOLD (Ethicon, Tokyo, Japan). The proximal and distal sides of the intestinal tract were transected using a 60‐mm linear stapler.

### 2.3 Procedure for OLA

**FIGURE 1:**
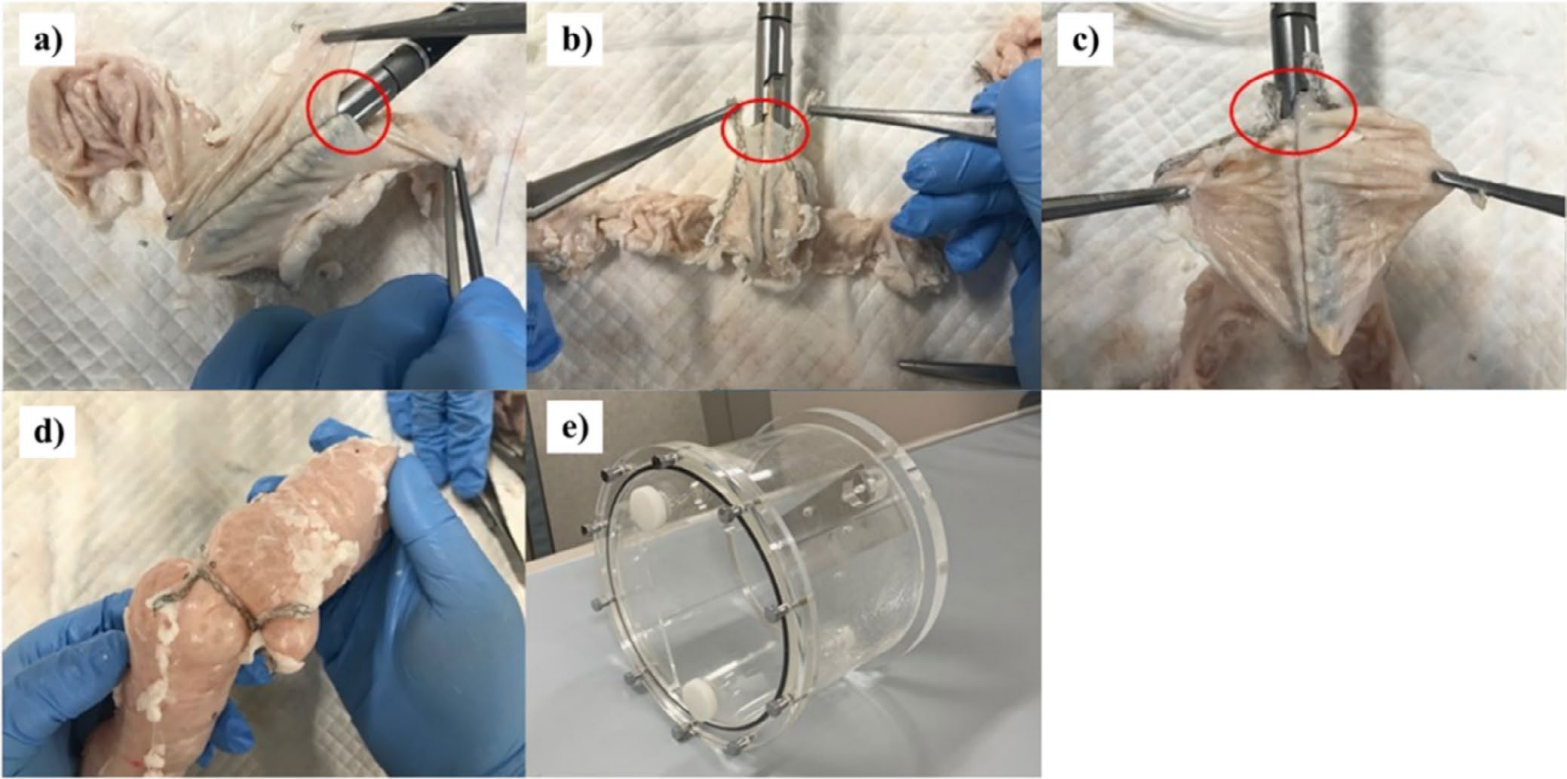
Procedure for each anastomosis and CT imaging protocol. (a) OLA, (b) DSA, and (c) FEEA. The red circles indicate enterotomies. (d) Air was infused into the models until intraluminal pressure was maintained at 20 mmHg. (e) CT QA Phantom JCT II (Kyoto Kagaku, Kyoto, Japan).

Figure 1a shows the procedure for OLA. This procedure was performed as previously described in the literature [13]. Enterotomies were performed on opposite sides of the mesentery. Isoperistaltic side‐to‐side anastomosis was performed using a 60‐mm linear stapler. Furthermore, four 3–0 standing stitches were applied along the enterotomy to uniformly lift its edges. A second 60‐mm linear stapler was used to close the enterotomy mechanically.

### 2.4 Procedure for DSA

Figure 1b shows the procedure for DSA. This procedure was performed as previously described in the literature [14]. Transverse enterotomies were performed at the mesenteric edges of the stapling line in both intestines. Each 45‐mm jaw of a 60‐mm linear stapler was inserted into the enterotomies along the posterior wall of both intestinal tracts and subsequently fired. Furthermore, four 3–0 standing stitches were applied along the enterotomy to uniformly lift its edges. The enterotomy was partially closed with the first 30 mm of a 60‐mm linear stapler and subsequently completely closed with the second 60‐mm linear stapler.

### 2.5 Procedure for FEEA

Figure 1c shows the procedure for FEEA. This procedure was performed with a reference to the literature [15]. Enterotomies were created at the antimesenteric edges of the stapling line of both intestines. Antiperistaltic side‐to‐side anastomosis was performed using a 60‐mm linear stapler. Furthermore, four 3–0 standing stitches were applied along the enterotomy to uniformly lift its edges. A second 60‐mm linear stapler was used to close the enterotomy mechanically.

### 2.6 Experiment 1

Three‐dimensional images of each anastomosis model were prepared for comparison. Additionally, the length of each staple line comprising the anastomosis site was measured for each model.

### 2.7 Preparation of Anastomosis Models

After anastomosis was completed, a 16‐Fr Foley catheter was placed into the lumen at the end of the intestine in each anastomotic model, and the balloon was inflated to close the lumen. A purse‐string suture was placed in the insertion hole around the catheter. A balloon catheter was connected to an inflation pump and sphygmomanometer. Air was infused into the models until the intraluminal pressure reached 20 mmHg. The deflated balloon catheter was removed, and the purse‐string suture was ligated. Figure 1d shows the anastomosis model with an internal pressure maintained at 20 mmHg.

### 2.8 Computed Tomography Imaging Protocol

Each anastomotic model was placed inside an acrylic cylindrical container with a diameter of 120 mm and filled with water. The cylindrical container was subsequently fixed in the center of a computed tomography (CT) QA Phantom JCT II (Kyoto Kagaku, Kyoto, Japan) with an outer diameter of 189 mm and filled with water for CT scanning (Figure 1e). A 64‐channel multi‐detector row CT scanner (Discovery CT750 HD; GE Healthcare, Waukesha, WI, USA) was used. The number of acquisition rows and slice thicknesses were 64 and 0.625 mm, respectively, with a 0.4‐s rotation time. The exposure conditions included 120 kVp with an automatic tube current modulation system and Auto mA installed in the CT scanner. All images’ matrix had a resolution of 512 × 512 pixels. Noise suppression processing, which is categorized as a nonlinear image quality improvement technique, was not used. Three‐dimensional reconstruction was performed using Ziostation2 software (Ziosoft, Tokyo, Japan). We compared each anastomosis in terms of the three‐dimensional shape, circumference, and length of each stapler line. The total circumference of the anastomosis was measured in volume rendering mode (general three‐dimensional display). The position where the anastomosis cross‐section appeared visually largest was selected, and the circumference at that point was measured. The position appearing visually largest was determined by two physicians (blinded to the type of anastomosis). In cases where there were discrepancies between evaluations, the two physicians discussed their assessments and conducted a re‐evaluation. If necessary, a third physician acted as an arbitrator to finalize the assessment. The staple lines configured for each anastomosis were linearized and measured. The length of the first stapler line indicates the length of the staple line suturing the wall of the intestinal tract. The length of the enterotomy closure refers to the length of the staple line(s) suturing and closing the enterotomy.

### 2.9 Experiment 2

**FIGURE 2:**
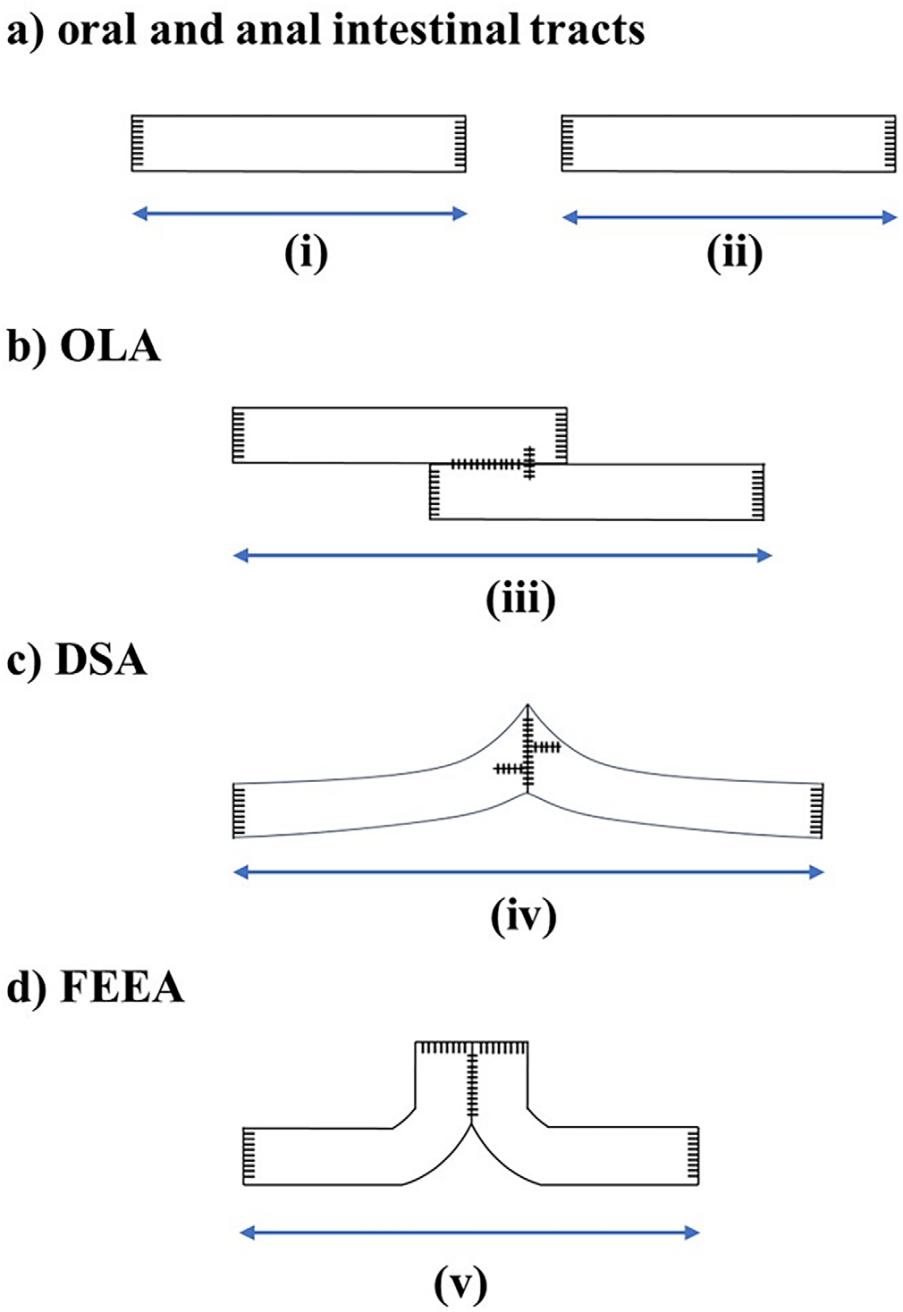
Findings from the comparison of shortened intestinal tract before and after anastomosis in each model. (a) The length of two intestinal tracts before anastomosis, referred to as (i) and (ii), was measured. The sum of (i) and (ii) is the total length of the two intestinal tracts. (b–d) After creating the anastomosis models, the total length of the models was measured (iii–v). The shortened length of the intestinal tract was defined as the difference between the sum of (i) and (ii) and the lengths of each anastomotic level (iii–v).

Findings from the comparison of shortened intestinal tract before and after anastomosis in each model are shown in Figure 2. The total length of the two intestinal tracts was measured before creating the model (Figure 2a). After creating each anastomosis model, the overall length of the intestinal tract was measured (Figure 2b–d). The shortened length of the intestinal tract was defined as the difference in length before and after anastomosis.

The combined lengths of the two intestinal tracts before anastomosis were compared with the length of a single intestinal tract after anastomosis for each anastomotic model.

### 2.10 Statistical Analysis

One‐way analysis of variance (ANOVA) was used to compare the means among different groups. ANOVA is a robust method for determining whether there are statistically significant differences between the means of three or more independent groups. To further analyze the specific differences between group pairs, the Tukey–Kramer multiple comparisons post‐test was employed. This test was chosen because it controls for the family‐wise error rate when making multiple comparisons, thus reducing the risk of Type I errors. All statistical analyses were performed using JMP Pro 16 software (SAS Institute, Cary, NC, USA), and statistical significance was indicated by a *p* value < 0.05.

## 3 Results

### 3.1 Experiment 1: Three‐Dimensional Imaging

**FIGURE 3:**
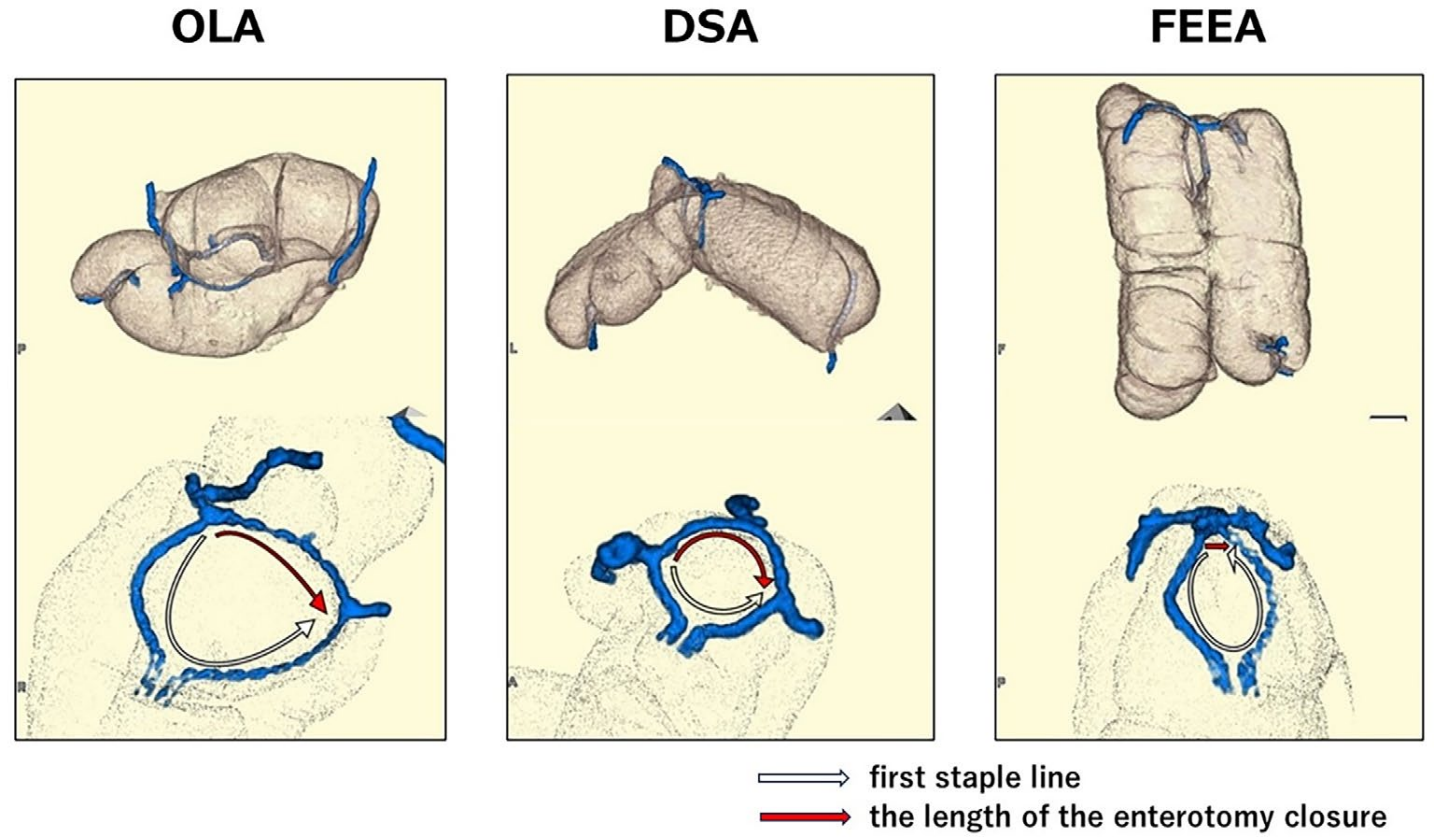
Three‐dimensional images of each anastomotic model. All staple lines are colored blue. White arrows show the first stapler line and red arrows show the length of the enterotomy closure.

**FIGURE 4:**
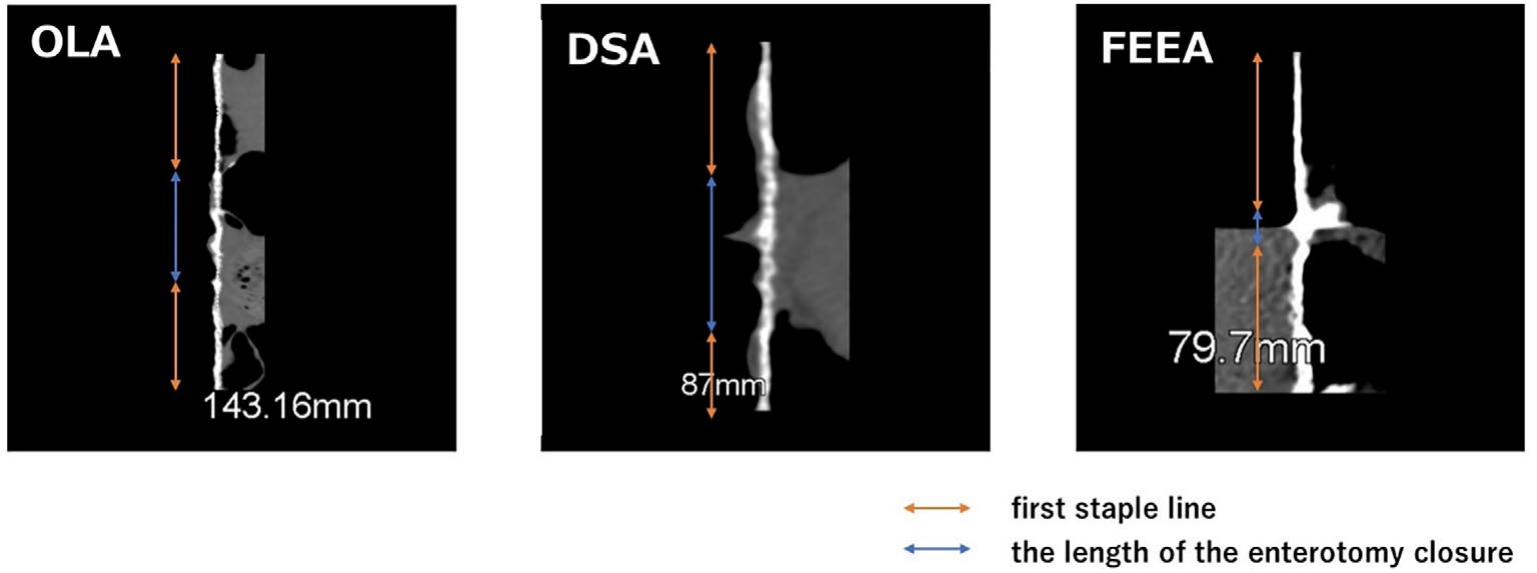
Linearized staple line comprising the circumference of each anastomosis area. Orange arrows show the first staple line of side‐to‐side anastomosis, and the blue arrows show the length of enterotomy closure.

The three‐dimensional shape of each anastomosis is shown in Figure 3 and Videos [Link], [Link], [Link]. The lengths of the staple lines comprising the anastomosis site, measured from the three‐dimensional shape for each anastomosis model, along with the number of staplers required and direction of peristalsis, are shown in Table 1. The circumference of the anastomosis in OLA (141.5 ± 3.3 mm) was significantly greater than that in DSA (87.9 ± 0.9 mm; *p* < 0.001) and FEEA (89.6 ± 10 mm; *p* < 0.0001). Figure 4 shows the linearized staple lines comprising the anastomotic site, categorized into the first staple line and the staple line(s) closing the enterotomy. Orange arrows show the first staple line, and blue arrows indicate the length of the enterotomy closure. The length of the first stapler line in DSA (55.7 ± 8.9 mm) was significantly shorter than that in OLA (98.1 ± 5.5 mm; *p* < 0.003) and FEEA (84 ± 11 mm; *p* < 0.02). The length of the enterotomy closure in FEEA (5.6 ± 1.2 mm) was significantly shorter than that in OLA (43.3 ± 6.7 mm; *p* < 0.001) and DSA (32 ± 8.9 mm; *p* < 0.006).

**TABLE 1:** Comparison of the staple lengths comprising the anastomosis site in each model and the number of staplers required.

	OLA (*n* = 3)	DSA (*n* = 3)	FEEA (*n* = 3)	*p*
Circumference of the anastomosis (mm)	141.5 ± 3.3	87.9 ± 0.9	89.6 ± 10.0	*p* < 0.001 OLA versus DSA, *p* < 0.0001 OLA versus FEEA
Length of the first stapler (mm)	98.1 ± 5.5	55.7 ± 8.9	84 ± 11	*p* < 0.02 DSA versus FEEA, *p* < 0.003 DSA versus OLA
Length of the enterotomy closure (mm)	43.3 ± 6.7	32 ± 8.9	5.6 ± 1.2	*p* < 0.001 FEEA versus OLA, *p* < 0.006 FEEA versus DSA
Peristaltic direction	Peristalsis	Peristalsis	Antiperistalsis	
Required number of the linear staplers	4	5	4	

Abbreviations: DSA, delta‐shaped anastomosis; FEEA, functional end‐to‐end anastomosis; OLA, overlap anastomosis.

### 3.2 Experiment 2: Intestinal Tract Shortening

Table 2 shows the shortened length of the intestinal tract after anastomosis in each model. The shortened length of the intestinal tract after anastomosis in DSA (33 ± 6.9 mm) was significantly shorter than that in FEEA (76 ± 2 mm; *p* < 0.0001) and in OLA (60 ± 5 mm; *p* < 0.002). The length in the OLA group was significantly shorter than that in the FEEA group (*p* < 0.02).

**TABLE 2:** Shortened length of the intestinal tract after the anastomosis in each model.

Group	*n*	Shortened length of the intestinal tract after anastomosis (mm)
OLA	3	60 ± 5*
DSA	3	33 ± 6.9**
FEEA	3	76 ± 2

Abbreviations: DSA, delta‐shaped anastomosis; FEEA, functional end‐to‐end anastomosis; OLA, overlap anastomosis.

* *p* < 0.02 versus FEEA.

** *p* < 0.002 versus OLA, *p* < 0.0001 versus FEEA.

## 4 Discussion

To the best of our knowledge, our study is the first study to develop a technique to visualize gastrointestinal anastomoses in three dimensions under conditions closely resembling those of the human body and to accurately measure the parameters of the anastomotic site. This allowed us to precisely measure the size of the anastomotic site and the length of intestinal mobilization required for anastomosis. By employing this technology to evaluate three IA models, we were able to recognize the advantages, limitations, and pitfalls of each IA technique.

Minimally invasive colectomy, using a laparoscopic or robotic approach, has become the standard procedure for benign and malignant colonic diseases in many centers worldwide [16, 17]. Additionally, owing to advances in surgical devices such as stapling devices, laparoscopic coagulating shears, and bipolar vessel‐sealing devices, IA, which is a more demanding procedure, has become easier to perform. Unlike EA, IA involves the extraction of specimens through a shorter Pfannenstiel incision, making it impossible for the surgeon to confirm the actual anastomotic site by touch during surgery. This inability to confirm the patency of the anastomotic site stresses surgeons. Currently, the only method for evaluating the anastomotic site in clinical practice is to identify the staple line using postoperative CT scans [18]. Experiments using anastomotic models to measure pressure resistance and investigate the anastomotic site using circular staplers after rectal surgery via colonoscopy [19, 20, 21]. However, no studies have focused specifically on or objectively evaluated the anastomotic site after colectomy. Given this background, we created three anastomosis models—OLA, DSA, and FEEA—under conditions closely resembling clinical settings and conducted two experiments to compare and examine each IA technique in detail [13, 14, 15].

In experiment 1, a three‐dimensional image of an IA model was created, allowing for visualization, precise measurement, and evaluation of the staple line. The intraluminal pressure of the human colon has been reported to be 10–20 mmHg [22, 23]. Water phantoms exhibit x‐ray absorption and scattering effects similar to those of the human body. In this experiment, the anastomotic model was placed inside an acrylic cylindrical container and subsequently inserted into a CT phantom for scanning. Without the acrylic cylindrical container, the buoyancy of the air inside the anastomotic model would cause it to float to the top of the CT phantom, making the model adhere to the wall of the CT phantom container. This adherence would complicate the construction of a three‐dimensional image using Ziostation, as the exterior of the CT phantom wall would be surrounded by air. By maintaining the intraluminal pressure of the anastomosis model at 20 mmHg and properly using a water phantom, it was possible to capture accurate CT images without artifacts under conditions almost identical to those of the human body.

In experiment 2, the shortened length of the intestinal tract after anastomosis was measured. Even in EA, it is impossible to safely perform anastomosis without adequate removal of the intestinal tract through a small abdominal incision. Although the range of intestinal mobilization required for IA is less than that required for EA [24], in left hemicolectomy or transverse colectomy, it may still be necessary to mobilize the hepatic flexure, splenic flexure, or both to ensure the minimum required length of the intestinal tract for safe anastomosis. The values measured in experiment 2 provided crucial information for selecting the IA method. Based on the results of these two experiments, the following considerations can be made for each of the three anastomosis techniques.

OLA requires the use of four staplers and is an isoperistaltic anastomosis technique. OLA was found to have the longest staple line compared with DSA and FEEA. This was attributed to the use of a 60‐mm stapler for side‐to‐side anastomosis. Additionally, unlike FEEA, which closes the first stapler vertically in the closing of the enterotomy, OLA uses a 60‐mm stapler to close the enterotomy horizontally, resulting in an anastomosis that approximates the shape of an equilateral triangle. The length of the first staple line in the OLA group was the longest at 98 mm. This was due to the minimal length of the staple line removed by closing the enterotomy; however, it was surprising that only a 98‐mm line was formed, despite the expectation for a 120‐mm staple line with the first 60‐mm stapler. The shortened length of the intestinal tract after anastomosis in the OLA was approximately 6 cm, indicating that the mobilization range required for anastomosis was longer than that required for DSA but smaller than that required for FEEA.

DSA is an isoperistaltic anastomosis technique that requires the use of five staplers with a total circumference of approximately 88 mm. In DSA, a 45‐mm stapler was used for side‐to‐side anastomosis, and only a 55‐mm staple line was formed. Contrastingly, the length of the staple line for closing the enterotomy with two staplers of 30 and 60 mm was the longest in DSA at 32 mm. Accordingly, in DSA, excessive resection of the first staple line using the second and third staplers may pose a risk of anastomotic stricture. However, the intestinal tract shortening after anastomosis was 33 mm, the smallest among the three techniques. This suggests that compared with the other techniques, DSA can be performed with minimal mobilization of the intestinal tract. Therefore, DSA may be an effective anastomotic technique for IA when the available intestinal length for anastomosis is limited.

FEEA is an antiperistaltic anastomosis technique that requires the use of four staplers with a total circumference of approximately 90 mm. FEEA is also the most frequently performed anastomosis technique and has the longest history of the three techniques [25]. However, the shortened length of the intestinal tract after anastomosis was 76 mm, which required the largest range of intestinal mobilization among the three techniques.

Our study suggests that OLA resulted in the largest anastomotic site. The reported incidence of anastomotic stricture after colorectal surgery ranges from 2% to 28%; however, no studies have specifically investigated the incidence of anastomotic stricture following IA [26, 27, 28, 29]. Risk factors for anastomotic stricture include tissue perfusion at the anastomotic site and anastomotic leakage, but the initial anastomotic circumference is also considered a crucial determinant for patency [28, 30]. In 2025, we reported the short‐term outcomes of intracorporeal OLA in laparoscopic colectomy using the same method as that used in this study [31]. Our previous study suggested that intracorporeal OLA may offer advantages, such as reduced blood loss, shorter hospital stays, smaller incisions, and lower inflammatory responses, over EA. Furthermore, no cases of anastomotic stricture were observed with the use of OLA in that study. Notably, to date, there have been no reports on the optimal anastomotic area for colo‐colonic or ileo‐colonic anastomosis. Further studies are required to evaluate the clinical implications of the anastomotic circumference demonstrated in this study. Notably, this study suggests that among the three techniques, DSA can be performed with the least bowel mobilization. In cases where securing a sufficient intestinal length for anastomosis is challenging due to anatomical factors such as obesity or adhesions, DSA may be a beneficial option. Moreover, among the three techniques, DSA had the smallest anastomotic site. Importantly, side‐to‐side anastomosis was performed using a 45‐mm stapler in this study, in accordance with previous reports. While using a 60‐mm stapler might have resulted in a larger anastomotic site, it would have also increased the length of intestinal shortening. In contrast, in terms of cost, FEEA and OLA require four linear staplers to transect the oral and anal sides of the intestine, whereas DSA requires five linear staplers. The additional staple reloading costs range from $100 to $2000, depending on the brand and different financial agreements between the providing company and respective hospital administration. In Japan, the number of staples that can be used within the insurance coverage for colectomy is limited to four; hence, selection of FEEA or OLA may be preferred. Further investigation is needed to determine whether using four staplers in DSA could serve as an alternative to the conventional method that uses five staplers.

This study had some limitations. The pig intestinal tract was used in the experiment, which may not accurately reflect measurements in human intestines. In humans, the type of peristalsis, innervation, neighboring structures, and pressure from these structures certainly influence the function in vivo. Therefore, the results of this experiment cannot be fully applied to the human body. In cases of ileocecal resection or right hemicolectomy, ileocolic anastomosis was performed. For intestinal anastomosis, it is essential that the inlet, anastomotic site, and outlet maintain sufficient patency. In DSA and FEEA, the anastomosis procedure had a minimal impact on the patency of the inlet and outlet. However, caution should be exercised while using the OLA technique. When closing the enterotomy in OLA, part of the intestinal wall near the enterotomy is resected, which could affect the patency of either the inlet or outlet. This could directly influence the width of the anastomotic site. In this study, although no obvious stricture was observed in the three‐dimensional anastomosis model of OLA, the patency of the inlet and outlet could not be evaluated. This limitation may affect the clinical outcomes. Furthermore, the sample size of the anastomosis models was three for each model. While we considered that individual differences in the intestines used to create the anastomosis models would have a minimal impact on the experimental results, analyzing a larger sample size might have been preferable. Additionally, for the measurement of each anastomotic site, objective metrics such as automated or software‐based measurement tools were not utilized, and the cross‐section of the anastomotic site was determined by three physicians. Given the small sample size, observer bias in these measurements cannot be completely ruled out. Finally, IA procedures in colectomy are continuously evolving; the procedure used in this experiment is based on prior reports, and even more refined IA techniques have been established since then.

In conclusion, we successfully visualized the staple line of the intestinal anastomosis three‐dimensionally for the first time, specifically focusing on IA after colectomy. This allowed for a detailed comparison of different anastomotic techniques. The results of this experiment suggest that the largest anastomotic site was created using the OLA technique, while minimal bowel length was required for the DSA technique. This research will be of great assistance to surgeons in selecting the appropriate anastomosis technique for IA. Future prospects in this field include using this technology to further advance the field of gastrointestinal surgery.

## Author Contributions

We confirm that all authors have read and approved the final version of this manuscript and agree to its submission. In accordance with the latest guidelines of the International Committee of Medical Journal Editors (ICMJE), the contributions of each author are quantified as follows. The contributions of the individual authors: Y.F. and S.S.: writing, editing, and revising the manuscript. Y.F., S.Y., S.K., S.S., and H.M.: conducting the experiments and collecting the data. H.T., Y.M., and K.K.: statistical analyses and reviewing the experimental design. S.T.: reviewing and editing the manuscript. All authors: provided feedback, added suggestions, or corrected previous versions of the manuscript and approved the final manuscript, sharing collective responsibility for its contents. All authors take full responsibility for the integrity and accuracy of the work.

## Ethics Statement

This study was approved by the Ethics Committee of the Kariya Toyota General Hospital (approval number: 658) and conducted in compliance with the ARRIVE guidelines. Additionally, all methods were performed in accordance with relevant guidelines and regulations.

## Consent

The authors have nothing to report.

## Conflicts of Interest

The authors declare no conflicts of interest.

## Supporting information


**Video S1.** Three‐dimensional video of DSA model. DSA, delta‐shaped anastomosis.

**Video S2.** Three‐dimensional video of FEEA model. FEEA, functional end‐to‐end anastomosis.

**Video S3.** Three‐dimensional video of OLA model. OLA, overlap anastomosis.

## Data Availability

The data that support the findings of this study are available from the corresponding author upon reasonable request.
